# Confronting genetic gains with markets: Retrospective lessons from New Rice for Africa (NERICA) in Uganda

**DOI:** 10.1177/0030727020948967

**Published:** 2020-08-20

**Authors:** Kofi Britwum, Eric S Owusu, Matty Demont

**Affiliations:** 1 School of Business and Professional Studies, 10671 Upper Iowa University, Fayette, IA, USA; 2 Agricultural and Resource Economics, 7712 University of Connecticut, Storrs, CT, USA; 3 Formerly at CSIR-Food Research Institute, Accra, Ghana; 4 63180 International Rice Research Institute (IRRI), Los Baños, Philippines; Formerly at Africa Rice Center (AfricaRice), Saint-Louis, Senegal

**Keywords:** NERICA, genetic gains, rice, experimental auctions, willingness to pay, Uganda

## Abstract

Breeders have two non-exclusive strategic investment options for increasing smallholder farmers’ and consumers’ livelihoods through genetic improvement of crop varieties: (i) enhancing productivity; and (ii) enhancing value and market access. New Rice for Africa (NERICA) varieties with superior agronomic characteristics were bred and introduced in various African countries in the early 2000s. Two decades later, drought tolerant NERICA4 is among the popular upland rice varieties grown across Africa. We analyze market evidence for NERICA4 from Uganda in 2011, i.e. well before it massively reached urban markets, where it is currently commingled with standard rice. We then compare the breeding priorities that would have ensued from the 2011 market evidence with the reality a decade later. Non-hypothetical auction experiments with consumers were conducted in urban Uganda in 2011 to predict potential market share and value of non-fragrant NERICA4 and fragrant NERICA1, relative to two market standards, i.e. non-fragrant *Kaiso*, and *Supa*, the most popular fragrant rice variety in the region. Average consumer bids positioned the two NERICAs between both market standards. Whereas NERICA1 easily outcompeted NERICA4 and *Kaiso* in the non-fragrant rice category, it failed to compete with *Supa* in the fragrant category. The 2011 market evidence would have suggested breeders prioritize investment in breeding programs for fragrant NERICAs to help smallholders gain access to high-value markets and expand consumers’ choice with cheaper fragrant rice alternatives. However, the popularity of NERICA4 relative to NERICA1 in farmers’ fields seems to suggest that agronomic genetic gains may have outweighed market traits such as fragrance.

## Introduction

As with many strategic agricultural investments targeted at enhancing smallholder farmers and consumers’ livelihoods through genetic improvement of crop varieties, the goals of breeders have been two-fold: (i) enhancing productivity; and (ii) enhancing value and market access. In line with this, New Rice for Africa (NERICA) varieties with superior agronomic characteristics were bred and introduced in various African countries in the early 2000s. Blending traits from the African traditional and Asian varieties, NERICA varieties have superior agronomic characteristics such as better weed resistance, resilience against major African biotic and abiotic stresses, high fertilizer returns, and high yields (Wopereis et al., 2008). The popularity of NERICA across several regions of sub-Saharan Africa (SSA) has been staggering; about 700,000 hectares of NERICA varieties were estimated to have been cultivated in 2009, increasing to 1.4 million hectares in 2013 by 1 million farm households (Chuhan-Pole and Angwafo, 2011; Diagne et al., 2010; Rice Hub, 2020). The socioeconomic gains from NERICA adoption have also been impactful, with evidence of significant poverty alleviation successes among planters of the varieties (Arouna et al., 2017; Kijima et al., 2008).

Despite the advantages of NERICA and its potential to meet governments’ objective of boosting local rice production, initial adoption among farmers in Uganda was mixed; a significant number of early adopters gave up cultivation (Kijima et al., 2011). The moderate adoption was at the time pinned to restricted access to NERICA seeds and a challenging start to the implementation of the NERICA varieties’ program (Kijima et al., 2011). However, one NERICA variety, NERICA4, successfully managed to become one of the most popular upland rice varieties grown across Africa. NERICA4 is drought tolerant, has an early maturity, and is high yielding. These outcomes, thus, highlight the need to not only examine supply chain linkages for new crop varieties (Kijima et al., 2011), but to also integrate demand driven interventions, without which value chain developments could be hampered (Demont, 2013).

In Uganda, NERICA varieties were introduced in early 2002 (Fujiie et al., 2010; Kitanaka et al., 2009) and in 2015, the seeds of these varieties managed to gain the highest market share in the country, i.e. around 22% of all rice seed produced (Barungi and Odokonyero, 2016: 16). Similar to other countries, NERICA4 has been the most successful NERICA variety in Uganda, an outcome attributed to its drought and disease resistant properties (MAAIF, 2019; Matsumoto et al., 2014). A decade and a half after their introduction, it is time to draw retrospective lessons from NERICA to generate insights on the optimal breeding strategies for increasing smallholder farmers’ and consumers’ livelihoods through genetic improvement of crop varieties. Was the success due to increased smallholder productivity given improved agronomic traits, or the result of expanded access to urban markets driven by highly demanded market traits in the region such as, for example, fragrance (Mgendi et al., 2018)?

Defining the right breeding priorities for rice is of utmost importance, being a global staple for over half the world’s population with an exponential growth in consumption across many countries in SSA. Indeed, having recorded more than a 50% increase in per-capita rice consumption within these last decades, growth in SSA has been the fastest anywhere in the world (Mohanty, 2013). This increase has been driven by factors such as a growing population, dietary transitions, and convenience associated with cooking rice relative to other staples (Muthayya et al., 2014; Seck et al., 2012; Stryker, 2013). The questions raised are particularly important for Uganda, because consistent with the general trends in SSA, rice is growing in popularity in Uganda. It is not a traditional staple, but its growing prominence in urban Ugandan diets has been driven by some of the factors previously mentioned (Haggblade and Dewina, 2010). To meet rising demand, the country runs a high rice import bill; by some estimates this could average $90 million annually (Africa Rice Center, 2006). Aimed at cutting the rising imports of the staple and the consequent pressure on foreign reserves, the Ugandan government is currently developing a new National Rice Development Strategy (NRDS) under the Phase 2 of the Coalition for African Rice Development (CARD) to revamp local rice production with the goal of attaining self-sufficiency by 2030 (CARD, 2020).

Since their release, NERICA varieties have gradually reached urban markets in Uganda, where they currently tend to be sold unbranded and commingled with the variety *Kaiso*, which has become a generic brand name for standard, non-fragrant rice by Ugandan consumers (Kikuchi et al., 2013). Hence, since the arrival of NERICAs on the market, the market standard *Kaiso* has gradually become a mixture of NERICAs and older upland varieties, which renders it difficult to use *Kaiso* as a market reference for comparison purposes. Commingled NERICA and *Kaiso* are further blended with *Supa*, the premium fragrant rice variety on the market which is most popular in the region (see Kikuchi et al., 2013). Consumers have now become familiarized with the new blended market standards. In order to analyze pure consumer response to NERICAs, relative to the original *Kaiso* and *Supa* benchmarks, it is important to go back in time and identify market evidence for NERICAs shortly after their release and before they massively reached urban markets.

In 2011, the Africa Rice Center (AfricaRice) collected data based on controlled, non-hypothetical auction experiments with consumers in urban Uganda to predict potential market share and value of fragrant and non-fragrant NERICA varieties, relative to the two prevailing market standards *Kaiso* and fragrant *Supa* (Demont and Ndour, 2011). Specifically, the study examined preferences for the non-aromatic NERICA4, and the relatively aromatic NERICA1 variety. While both have superior agronomic characteristics, fragrance is a distinguishing attribute. In light of this, meaningful comparisons can be drawn between the popularly known and cultivated fragrant rice, *Supa*, and the new NERICA1 bred as a fragrant variety. This dataset provides a unique opportunity to analyze the drivers of consumers’ willingness to pay (WTP) for NERICA varieties, relative to the two non-commingled market standards, and compare the retrospective breeding priorities that would have ensued from the 2011 evidence with the reality a decade later. For example, is the current success of NERICA4 relative to NERICA1 in terms of adoption in smallholder farmers’ fields consistent with the urban demand for both varieties that was predicted in 2011?

The contribution of this paper is to draw lessons in terms of breeding priorities based on a retrospective look at NERICAs in Uganda. Most of the literature on the impact of NERICAs focuses on the supply side (productivity), while to the best of our knowledge none of the studies has looked at the demand side. The novelty of our approach is that we are able to confront the supply side with the demand side a decade after market evidence was collected. This retrospective look enables us to draw important lessons for trait prioritization in breeding. This proves to be a worthwhile exercise because we find a discrepancy between what the 2011 evidence would have suggested to breeders and the reality a decade later.

## Materials and methods

### Experimental methods and sampling

#### Location and setup

To achieve the study’s objective, a dataset from framed field experiments that were conducted in October 2011 was employed. Women shoppers, estimated to be at least 18 years were targeted in *Nakawa* market, a produce market in Uganda’s capital, Kampala. These potential subjects were first randomly approached by the research team with a flyer that displayed pictures of previous research experiments, and were asked whether they would be willing to participate in a 3-hour market research project and earn 25,000 USh [Ugandan Shillings (US$9.80); average exchange rate in 2011 at the time of the experiments was US$ 1 to 2,550 USh] in compensation. Those who consented to participate were transported to a hotel located 1 km (0.62 miles) from the market, the venue of the experiments. A total of 120 participants were recruited for the study.

#### Experimental design

Four different rice varieties were anonymously displayed in plain white bags at the front of the experimental venue (a conference room): (i) *Kaiso*, (ii) *Supa*, (iii) NERICA1 and (iv) NERICA4 (Demont and Ndour, 2011). These varieties differed only in their bundle of quality attributes, which was essential for the “endow-and-upgrade” design used in the study. Similar to Roosen et al. (1998), *Kaiso* rice was the designated benchmark variety, widely regarded to be inferior in quality to other varieties, and thus, allowing WTP to be elicited when upgraded to a higher quality. Being a common variety in the *Nakawa* market, *Kaiso* is easily recognizable as having short size, broken grain and admixtures e.g. dirt and husks, as it is usually poorly milled. Kaiso is non-fragrant, has a high swelling capacity when cooked, and is popular among low-income consumers (Ayoki, 2012). Supa is known for its fragrance, cleanliness, and was and still is considered as a premium upper benchmark. Both NERICAs have a long, intermediate grain size that are soft and chalky. While NERICA1 is noted for its aroma as previously noted, NERICA4 has been rated sticky and clean with a larger swelling ratio than NERICA1 (Futakuchi et al., 2013; Masette et al., 2013). As a result, the difference between (ii) and (i) revealed WTP for fragrance (and other grain quality and texture characteristics of *Supa* not present in *Kaiso*); the difference between (iii) and (ii) revealed competitiveness of NERICA1 relative to the market standard *Supa* within the fragrant rice category; the difference between (iv) and (i) revealed the competitiveness of NERICA4 relative to the market standard *Kaiso* within the non-fragrant rice category; and the difference between (iii) and (iv) revealed the relative competitiveness of both NERICAs across fragrance categories on the market. The characteristics and prices of the four rice varieties are summarized in Table 1.

**Table 1.** Characteristics and price of the four rice varieties.

**Variety**	**Characteristics**	**Average price/kg in 2011**
*Kaiso*	Non-fragrant; high level of brokenness; high swelling capacity; poorly milled; presence of admixtures	3,000 USh
NERICA4	Non-fragrant; intermediate grain; soft and chalky; sticky; clean; larger swelling capacity	3,190 USh*
NERICA1	Fragrant; intermediate grain; soft and chalky	3,190 USh*
*Supa*	Fragrant; clean; sticky; dominant in Nakawa market	3,800 USh

* Prices for NERICA1 and NERICA4 reflect 2011 production and transportation costs, not market prices.

The second-price Vickrey auction (1961) was chosen, given its demand revealing properties (see for example, Alfnes and Rickertsen, 2003; Lusk and Shogren, 2007) and its advantage of inducing subjects to bid their true WTP for auctioned products. Following Melton et al. (1996) and Roosen et al. (1998), three auction rounds were conducted.

A trial auction round was first conducted using a commonly known brand of biscuits (cookies) to help familiarize participants with the auction process. Endowed with a small pack of Riham milk biscuits, each participant was asked how much they would be willing to pay to upgrade to two superior biscuits, Britania Milk biscuits and Britania Glucose biscuits. With the research team satisfied with participant understanding of the auction mechanism in the training session, each participant was endowed with 1 kg of the benchmark rice (*Kaiso*) and was asked to choose between the benchmark and an alternative rice type. For each of the three alternative rice types, participants indicated their preferences between them and *Kaiso*. Those who chose the alternative over the benchmark were requested to submit their WTP bids to upgrade *Kaiso* to the “superior” alternative. Only participants who opted for the alternative rice over the benchmark were asked their WTP to upgrade. For those who chose *Kaiso even if it was priced the same as* the alternative, a WTP of zero was recorded with no opportunity to upgrade. Bids were submitted for all three alternative rice types.

It was explained that only one product and one bidding round would be binding, a strategy intended to incentivize participants bidding their true values in each round. Sensory impacts of the rice varieties on the likelihood of upgrading and WTP were examined next. These were designed to allow participants to experience the quality attributes—aroma (fragrance), taste, texture, and stickiness—and swelling capacity of cooked rice for the three alternative varieties. An additional advantage of the sensory test—as with other procedures that have required consumption—was that it further contributed to minimizing hypothetical bias (similar to Britwum and Bernard, 2018; Lusk et al., 2004). In between tasting, participants cleansed their palates with water and then submitted WTP bids for the alternative rice types, again, with *Kaiso* as the benchmark.

As a final step, a common WTP bid agreed upon by participants in groups of fives were elicited, to upgrade each alternative variety from *Kaiso*. Also known as the collective induction treatment (CIT), this was included to determine the influence of social cognition on rice preferences (Akoa et al., 2016; Demont et al., 2013a, 2017). Following the CIT, participants *individually* submitted final WTP bids for each of the three rice types. In total, eight experimental sessions were conducted over the course of 4 days, with two sessions conducted per day. Data on participants’ socio-demographic characteristics were gathered using a survey after the first bidding round. A second survey was administered post-CIT to collect information about preferences and knowledge of the rice types. Information on the full experimental protocol is published in Demont and Ndour (2011).

#### Sample of participants

Summaries of participants’ attitudes and knowledge, some design-specific variables, and demographics are presented in Table 2. Participants’ awareness of NERICA was relatively low. Just about a third of participants had expressed some familiarity about the variety. Rice fragrance appeared to be an important attribute though among participants, with about 90% of them preferring fragrant rice. This is consistent with the general preference for aromatic rice in Eastern Africa and, more particularly in Tanzania (Mgendi et al., 2018), from where the aromatic rice variety *Supa* originates. With respect to the experimental design, half (50%) of the auctions were conducted in the morning. A sizable majority (68%) self-reported to be hungry at the time of the experiments.

**Table 2.** Variable names, definition, and descriptive statistics.

**Variable**	**Description**	**Mean**
Attitudes and knowledge		
Awareness	1 if subject was aware of the NERICA varieties; 0 otherwise	0.38
Preference for fragrance	1 if subject prefers fragrant rice; 0 otherwise	0.91
Field experiment		
Morning auctions	1 if experiment was conducted in the morning; 0 otherwise	0.50
Hungry	1 if subject was hungry during the sessions; 0 otherwise	0.68
Demographics		
Trader	1 if subject is a trader; 0 otherwise	0.38
Housewife	1 if subject is a housewife; 0 otherwise	0.11
Operates restaurant	1 if subject operates a restaurant; 0 otherwise	0.13
Social group	1 if subject belongs to a social group; 0 otherwise	0.42
Housemaid	1 if subject has a housemaid; 0 otherwise	0.10
Cooking hours	Total monthly cooking hours	175.9 *80.1*
Age	Age in years	36.8 *2.5*
Income	Monthly household income in thousand USh	312.9 *276.3*
Household size	Number of people living in household	5.6 *2.3*
Education		
<High school	1 if less than high school; 0 otherwise	0.52
Up to high school	1 if up to high school; 0 otherwise	0.43
Some college or higher	1 if some college or higher; 0 otherwise	0.05
Ethnic affiliation	1 if subject is from ethnic group; 0 otherwise	
Baganda		0.48
Banyagkole		0.09
Lango		0.08
Teso		0.07
Acholi		0.06
Busoga		0.05
Samia		0.03
Mugisu		0.03

*Note*: Numbers in italics are standard deviations.

Close to 4 in 10 were traders by vocation. Although just a few of the participants (11%) were housewives, only 1 in 10 had a housemaid. Also, a small number of participants (13%) were restaurant operators and a considerable proportion (42%) were members of a social group. Hours spent cooking rice in a typical month were quite substantial among participants (176 hours), a characteristic which rendered their opinions about new rice varieties essential. The average age of a participant was 37 years with average monthly household income at approximately USh 300,000 (which averaged to about US$117.65 in 2011). Average household size was about 6 members. Education levels ranged from low to moderate, with the highest education levels for over half the participants being elementary. A little over 40% of participants had completed high school education, while about 5% had attained some tertiary education. Demographic statistics included subjects’ ethnic affiliation; about half of study participants identified with the Baganda ethnic group.

## Econometric model

In the endow-and-upgrade framework, an individual is presented the choice to upgrade from a benchmark rice to an improved type in each bidding round. Conditional on the individual’s preferences, they first decide whether to upgrade or not, and then submit a bid consistent with their choice. It is assumed that the level of intrinsic and extrinsic rice quality, $q\in \left\{0,1\right\}$, characterizes preferences such that the indirect utility function for the utility-maximizing individual can be expressed as,

1 $$v^{q}\left(y;G\right)$$

where, *y* is income and *G* is a vector of observable attributes including those related to rice consumption (Carson and Hanemann, 2005; Hanemann, 1984). Prices have been dropped because they remain fixed in this setting. Faced with a valuation offer in a bidding round, the individual is assumed to compare utilities between the benchmark rice quality, $v^{0}\left(\cdot \right)$ and the alternative, $v^{1}\left(\cdot \right)$. In the case where they perceive the alternative variety as an improvement in intrinsic quality, the maximum amount they are willing to pay to attain this level of quality leaving them indifferent between the initial and final states of wellbeing is given by the identity (Bateman et al., 2002; Just et al., 2005),

2 $$v^{1}\left(y-WTP;G\right)=v^{0}\left(y;G\right)$$

The WTP for alternative (improved) rice quality can then be represented by the bid function,

3 $$WTP=f\left(y,G\right)$$

Reported WTP are expected to meet two theoretical restrictions: First, WTP must be bounded upward by the individual’s ability to pay, WTP≤y. Second, the individual either perceives alternative rice quality as an improvement or is indifferent, hence, WTP is non-negative, and restricted as 0≤WTP≤y.

Following Demont et al. (2013b), we estimate the bid function as a two-step process involving a first stage modeling of the desirability to upgrade (tier one), and a second stage estimation of actual bid submission conditional on choosing to upgrade (tier two). This is a typical corner-solution problem akin to the tobit setting, although we implement Cragg’s (1971) less restrictive but nesting framework in which the decision to upgrade and actual posting of bids are allowed to be governed by either separate or same processes (Burke, 2009). Letting $b_{ijs}$ and $w_{ijs}$ denote participant *i*’s desire to upgrade and submit bids, respectively, for product *j* in bidding round *s*, the first and second tier models are respectively given by the equations:

4 $$b_{ijs}=Z_{kijs}^{\text{'}}\gamma_{k}+e_{ijs};\;\;e_{ijs}∼N\left(0,\;1\right)$$

5 $$w_{ijs}=X_{lijs}^{\text{'}}\beta_{l}+\varepsilon_{ijs}$$

$$\begin{array}{l} b_{ijs}=1;\;w_{ijs}=w_{ijs}^{\text{*}}\text{ if}\;w_{ijs}^{\text{*}}>0\\ b_{ijs}=0;\;w_{ijs}=0\;\text{if}\;w_{ijs}^{\text{*}}\leq 0 \end{array}$$

where, $Z_{kijs}$ and $X_{lijs}$ are vectors of design-specific, preference-related, and individual-specific variables that determine the probability of upgrade and bid submitted in support of an upgrade, respectively; $\gamma_{k}$ and $\beta_{l}$ are the corresponding parameter vectors, and $e_{ijs}$ and $\varepsilon_{ijs}$ are the error terms, where the latter is assumed to be independently and identically distributed. Included in the vector of parameters are product dummies and interactions between product and awareness of NERICA. The log-likelihood function for equations (4) and (5) following Burke (2009) is expressed as,

6 $$lnL\left(\gamma ,\beta ,\sigma \text{ | }X,Z,w\right)=1\left(b=0\right)\;ln\left\{1-\text{Φ}\left(Z\gamma \right)\right\}+1\left(b=1\right)\text{ ln}[\text{Φ}\left(Z\gamma \right){\left(2\pi \right)}^{-\frac{1}{2}}\sigma^{-1}\text{exp}\{-{\left(w-X\beta \right)}^{2}/2\sigma^{2}\}/\text{Φ}\left(X\beta /\sigma \right)$$

where, $\text{Φ}\left(\cdot \right)$ is the cumulative distribution function of a standard normal and *σ* is the standard deviation.

Since each participant had multiple observations from different bidding rounds, the model was cluster corrected to yield robust standard errors. Similar to Newman et al. (2003), and Dong et al. (2004), the probability of an upgrade was modeled as a function of non-economic factors. As such, variables such as income, household size, and education were excluded from the first equation. The model also accounted for heteroskedasticity of income, given the likelihood of wide variances in income among our sample. While the remaining variables were included in the variance portion as well to test heteroscedasticity, those retained in the model (income) were variables that emerged statistically significant.

### Hypotheses

Expectations a priori for the study were largely guided by previous literature. First, it was deemed uncertain whether the experience of taste and other sensory characteristics and social cognition of the NERICA varieties would influence WTP. Previous studies have found varying responses to food products after sensory experience and social cognition (Akoa et al., 2016; Demont and Ndour, 2015; Demont et al., 2012; Lund et al., 2006; Nalley et al., 2006). As a result, preferences and WTP for the alternative rice varieties following both rounds were anticipated to be mixed, with the likelihood of a weak or strong preference depending on perceptions of them after tasting. Being aware of NERICA varieties was expected to increase preferences and/or WTP for these new varieties. Demont et al. (2013a) observed that awareness of an enhanced rice variety increased the likelihood of purchase among Senegalese consumers, although this did not significantly impact WTP. The impact of demographics on preferences and WTP were not known with certainty, although a few were intuitive. Participants with larger households, for instance, were expected to have lower WTP, largely due to economic concerns, while those with more income were anticipated to have higher WTP for the alternative rice varieties.

## Results

### Descriptive statistics

#### Willingness to pay for alternative rice, by rounds

Prior to determining factors that influenced subjects’ probability of upgrading, and their WTP for the alternative rice varieties, the WTP bids submitted in the different rounds and across rice alternatives were examined. Since the auction procedure was based on the endow-and-upgrade method, all WTP estimates have to be interpreted as the price premiums (or price discounts if negative) consumers are willing to pay on top of their perceived value of the benchmark variety *Kaiso*. Table 3 shows the percentage of zero bids and mean WTP disaggregated by product in the pooled case and by bidding round. Overall, only 18% of participants were *unwilling* to upgrade to *Supa*. However, about twice as many, 36% and 32%, had non-positive valuations for NERICA1 and NERICA4, respectively. With more participants willing to upgrade, *Supa* recorded the highest average WTP of 866 USh/kg (US$ 0.34). This indicates a greater preference for fragrant rice due to varietal and associated preference spillover from Tanzania (Mgendi et al., 2018). As of the experimental period, *Kaiso* was sold for 3,000 USh/kg (US$ 1.18) and *Supa* for 3,800 USh (US$ 1.49) (see Table 1). The observed price premium for *Supa* over *Kaiso* (866 USh/kg) thus validates participants’ WTP and the experimental process, as this premium closely matched the external market premium for *Supa*.

**Table 3.** Percentage of zero bids and mean WTP by product and bidding round.

**Product**	**Pooled**		**Pre-tasting**		**Post-tasting**		**Post-CIT**	
	**% (WTP = 0)**	**Mean (WTP > 0)**	**% (WTP = 0)**	**Mean (WTP > 0)**	**% (WTP = 0)**	**Mean (WTP > 0)**	**% (WTP = 0)**	**Mean (WTP > 0)**
Supa	18.1	865.6	8.3	953.3	24.2	797.5	21.7	845.8
		*807.3*		*831*		*844.6*		*741.3*
NERICA1	36.1	522.2	26.7	585.8	40.0	481.7	41.7	499.2
		*605*		*591.6*		*593.4*		*629.1*
NERICA4	31.9	440.0	21.7	497.5	35.8	458.3	38.3	364.2
		*540.4*		*571.2*		*585.4*		*450.4*

*Note*: Numbers in italics are standard deviations.

Variations, however, existed across bidding rounds. For all three rice types, the percentage of zero bidders increased after tasting—from 8 to 24% for *Supa*, from 27 to 40% for NERICA1 and from 22 to 36% for NERICA4. *Supa*, however recovered some of its preference post-CIT with a reduction in zero bids by about 2 percentage points, underscoring the effect of word of mouth (WOM) social opinions on economic values. On the other hand, more participants still viewed NERICA varieties negatively post-CIT given the corresponding modest decreases in the number of zero bids by about 2 percentage points in each case.

For each of the three rice alternatives, mean positive WTP bids before the tasting round were higher than post-tasting. It appears then that tasting cooked rice depressed subjects’ WTP, in some cases significantly so. This uniform decline was observed in other rice auctions as well and can be explained through the benchmark gaining acceptance and value when it was purified and cleansed for preparation and more information about its sensory attributes was obtained (Demont and Ndour, 2015). In the case of *Supa* and NERICA1, Wilcoxon significance tests show that WTP was significantly different at the 5.4% level or lower for these two varieties before and after tasting. The results further highlight subjects’ higher preference for NERICA1 than NERICA4. While average post-sensory bids were lower for each of the three rice versions, the average bid for NERICA1 was only slightly less than the pre-sensory average for NERICA4. This appears to suggest that between the two newer varieties, NERICA1 could be more successful with consumers than NERICA4.

As explained, participants were randomly assigned to groups of five, where each group agreed on a collective bid for the alternative rice versions after the tasting round. Participants then submitted individual bids after the group rounds. This allowed preferences to be confirmed or re-aligned through social cognition, after which subjects submitted individual bids. For both *Supa* and NERICA1 varieties, average WTP after the collective induction process was higher than the average in the post-tasting round. This suggests that despite the taste evaluations, subjects were willing to bid higher on average after they were given the opportunity to exchange WOM on their experiences of these varieties. The increase for the *Supa* variety was not significantly different between these two rounds, post-sensory and post-CIT (Wilcoxon test *p*-value of 0.5309). Given that *Supa* is a well-known brand, it is likely that participants had strongly established preferences for them, with the collective induction process appearing to confirm such preferences, rather than amplify. Average bids for NERICA1 similarly increased between post-sensory and the post-CIT rounds, but the difference was not significantly different. While it appears subjects’ preferences had been formed for the NERICA1 variety after tasting, the modest increase in mean WTP following the group effort may be indicative of moderate consumer acceptance of them beyond the tasting experience, an outcome which can be construed as encouraging for developers of the NERICA variety.

The more surprising finding, however, was the outcome that bids after the collective induction round for NERICA4 were on average lower than the post-sensory bids, and significantly so (Wilcoxon test *p*-value of 0.0226). While mean post-sensory bids were lower for all three rice alternatives than pre-sensory, the lower mean WTP following the group rounds, relative to the post-sensory round (for NERICA4), may reveal some degree of uncertainty about the search attributes of this variety, which was further highlighted after the collective induction process. What is apparent here is that the collective induction round revealed more about preferences for all three rice versions than the pre-sensory and post-sensory rounds; the better known fragrant variety, *Supa*, was clearly the most preferred—as expected, given the general consumer preference for rice fragrance in this region (Mgendi et al., 2018)—with moderate acceptance for the NERICA varieties, particularly, NERICA1.

Previous literature has found varying consumer responses after tasting or sensory rounds. Demont and Ndour (2015) reviewed the literature on rice auctions in Africa and concluded that tasting almost invariably led to a decline in WTP for upgraded quality characteristics. Lund et al. (2006) similarly observed that even though preference for apples that had been stored over a shorter time frame declined after tasting rounds, the decline did not lead to a significant change in WTP. Nalley et al. (2006), however, noted a decline in bids after study participants tasted sweet potatoes. The auction literature about incorporating collective induction treatment to assess the impact of social cognition (WOM) on WTP is, however, scant. In collective induction, the expectation is that prevailing opinions about each rice variety following sensory evaluations will be reinforced through social cognition (Demont et al., 2013a). While Demont et al. (2017) found strong evidence for the fact that both sensory experience and social cognition reinforce WTP, Demont et al. (2012) and Akoa et al. (2016) did not find any evidence for the latter effect.

#### Awareness about NERICA and willingness to pay

**Figure 1.**
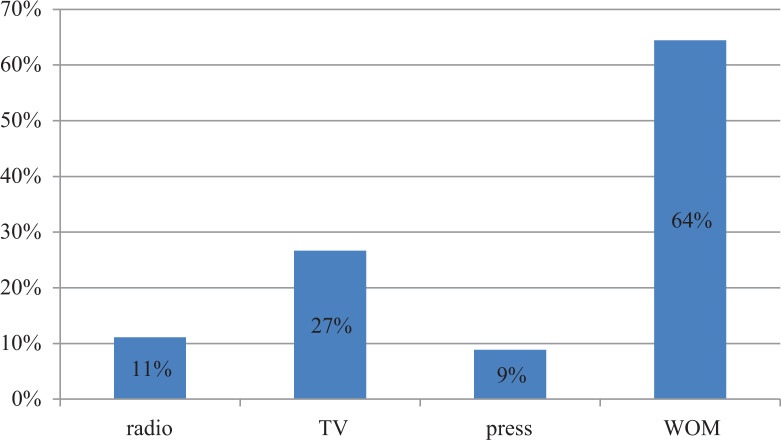
Sources of information received on NERICA rice (*n* = 45).

**Figure 2.**
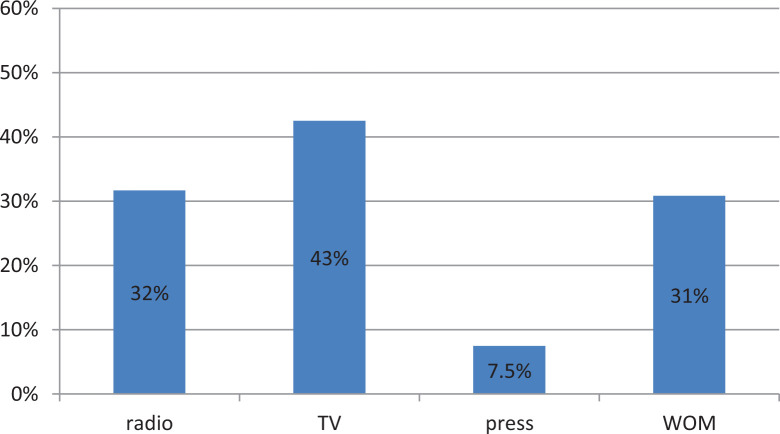
Most trusted sources of information used to judge the utility of a new rice type (*n* = 120).

Table 4 shows differences in WTP bids between participants who were aware of NERICA prior to the experiments, and those who were not. In the survey, participants were asked whether they were aware of the existence of NERICA. Also listed in the same question were other name variations that NERICA is known by such as *Suparica-2*, *Naric-3*, or *Bukenya*. As a result, we limit the definition of awareness in our study to a participant being aware of the existence of NERICA or the other name variations it was known by. For those who responded yes, they were asked to indicate where they received their information. As Figure 1 shows, less than half of those who had heard about NERICA indicated hearing about the variety from formal channels such as radio, television, or the press. Although formal information channels were the most trusted source of information (Figure 2), awareness of NERICA seems to be primarily generated through WOM communication. As hypothesized, WTP was higher in both pre-sensory and post-CIT rounds for NERICA1, compared with the post-sensory round, among participants who had prior knowledge of these varieties than those who did not. In the post-sensory round, however, those who had prior knowledge on average, submitted lower WTP bids than those who did not. Even though this was unexpected, it follows previous outcomes where participants discounted WTP after tasting (Demont and Ndour, 2015; Nalley et al., 2006). These differences were, however, not statistically significant.

**Table 4.** Mean WTP (USh) for NERICA varieties by status of NERICA awareness and bidding round.

	**NERICA1**			**NERICA4**		
	**Pre-tasting**	**Post-tasting**	**Post-CIT**	**Pre-tasting**	**Post-tasting**	**Post-CIT**
Aware of variety	644.4	453.3	568.9	437.8	306.7	340.0
	*590.7*	*587.6*	*681.5*	*591.0*	*376.2*	*376.8*
Unaware of variety	550.7	498.7	457.3	533.3	549.3	378.7
	*593.3*	*600.1*	*596.2*	*560.0*	*666.5*	*491.1*

*Note*: Numbers in italics are standard deviations.

Average WTP for NERICA4 among participants who had some knowledge about the NERICA varieties was lower in all rounds, relative to those who did not have any prior knowledge before the experiment. While these lower differences (between those aware and unaware) were not statistically significant for the pre-sensory and post-CIT rounds, it was for the post-sensory round at slightly lower than the 10% level of significance. Again, this suggests that consumer uncertainties about NERICA4 variety tended to be more pronounced after sensory experience, and more so among those who had some prior knowledge about the NERICA varieties. Overall, awareness of NERICA did not appear to influence preferences and WTP substantially. However, it supports the observation that consumers may be more open and accepting of NERICA1 than NERICA4 when they are familiar with NERICA. As noted, awareness was defined more broadly, capturing knowledge about the existence of NERICA, and should thus be interpreted cautiously.

### Results: Double-hurdle model

Results from the double-hurdle model, for the participation equation (desirability to upgrade) and the purchase equation (WTP bids) are displayed in Tables 5 and 6, respectively. In Table 6, three variants of the model are presented: Model [1] has no interaction; Model [2] has interaction between product and experimental round; and Model [3] features an interaction between product and NERICA awareness in addition to the product-round interaction. Recovered price premiums (discounts) based on Models [2] and [3] are presented in Table 7. See Appendix for approaches used in recovering marginal WTP values. There were 1,080 observations, comprising data from the three rounds (pre-sensory, post-sensory, and post-CIT), and the three rice types—*Supa*, NERICA4, and NERICA1—across the study’s 120 participants.

**Table 5.** Tier one of two-part model: Desirability to upgrade from benchmark to alternative rice.

	**Coefficient**	**Std error^#^**	**Marginal effect**
Constant	0.588	0.361	
NERICA4	−0.569***	0.111	−0.165***
NERICA1	−0.499***	0.129	−0.144***
Pre-tasting	0.537***	0.119	0.155***
Post-CIT	−0.069	0.094	−0.020
Attitudes and knowledge			
Awareness	−0.019	0.145	−0.006
Preference for fragrance	0.196	0.225	0.057
Field experiment			
Morning auctions	0.259*	0.133	0.075*
Hungry	−0.243*	0.144	−0.070*
Demographics			
Trader	0.291*	0.163	0.084*
Housewife	0.287	0.255	0.083
Operates restaurant	−0.059	0.239	−0.017
Social group	−0.118	0.134	−0.034
Housemaid	−0.465**	0.232	−0.135**
Cooking hours	0.001	0.001	0.0002
Age	0.005	0.007	0.001
Ethnic affiliation			
Baganda	−0.239	0.194	−0.069
Banyagkole	−0.072	0.264	−0.029
Lango	−0.015	0.249	−0.004
Teso	−0.263	0.292	−0.076
Acholi	0.640**	0.294	0.185**
Busoga	−0.604**	0.297	−0.175**
Samia	−0.864**	0.432	−0.250**
Mugisu	−0.207	0.432	−0.060
*N*	1,080		

^#^Robust standard errors which are cluster-corrected.

*p < 0.1; **p < 0.05; ***p < 0.01.

**Table 6.** Tier two of two-part model: WTP for alternative rice under three model scenarios.

	**[1]**		**[2]**		**[3]**	
	**Coefficient**	**Std error**	**Coefficient**	**Std error**	**Coefficient**	**Std error**
Constant	1257.9**	578.7	1227.9**	579.1	1144.0**	594.9
NERICA4	−739.1***	158.2	−590.5***	180.5	−420.1**	164.6
NERICA1	−597.4***	138.3	−694.1***	176.7	−680.9***	200.7
Pre-tasting	−84.6	81.1	−64.6	81.3	−67.5	82.2
Post-CIT	93.6	87.5	113.0	113.8	112.4	113.9
NERICA1 × Pre-tasting			121.9	122.7	125.2	125.5
NERICA1 × Post-CIT			166.6	158.8	163.8	159.7
NERICA4 × Pre-tasting			−200.6	151.6	−191.3	150.0
NERICA4 × Post-CIT			−256.1*	154.6	−239.6	148.4
Attitudes and knowledge						
Awareness	306.4*	164.4	307.4*	163.3	438.9**	224.1
Awareness × NERICA1					−35.5	189.0
Awareness × NERICA4					−484.4*	264.8
Preference for fragrance	-23.7	359.2	16.0	360.2	12.1	360.7
Field experiment						
Morning auctions	587.4***	178.2	586.2***	177.9	588.6***	178.9
Hungry	−118.3	222.3	−113.6	221.4	−108.3	222.4
Demographics						
Trader	−103.7	189.6	−108.3	188.7	−119.4	195.1
Housewife	170.2	260.3	162.1	260.2	145.9	258.4
Operates restaurant	−58.2	296.5	−63.8	292.2	−67.0	290.5
Social group	191.9	284.3	185.6	282.7	166.2	284.1
Housemaid	−171.9	311.1	−167.8	309.3	−207.5	332.5
Cooking hours	−1.2	1.0	−1.2	1.0	−1.2	1.0
Age	1.5	10.2	1.9	10.1	2.9	10.7
Income	−2.1	1.6	−2.1	1.9	−2.1	1.6
Income squared	−0.001	0.003	−0.001	0.003	−0.001	0.003
Household size	−121.7*	67.1	−122.6*	67.1	−123.8*	66.9
<High school education	−395.6	336.8	−496.7	335.0	−499.5	331.2
Up to high school education	−601.7	373.7	−602.3	372.6	−606.0	369.0
Ethnic affiliation						
Baganda	937.6***	335.3	936.2***	334.9	941.4***	337.6
Banyagkole	857.1**	406.8	864.0**	406.6	871.5**	412.6
Lango	709.5	450.2	709.0	449.0	694.0	442.5
Teso	69.6	477.8	77.2	473.7	74.5	462.3
Acholi	1610.2**	734.2	1606.3**	731.9	1579.6**	721.8
Busoga	439.2	434.3	440.6	432.8	432.4	439.2
Samia	1009.5*	534.0	1010.8*	535.8	993.3*	537.9
Mugisu	673.2	1177.2	667.4	1167.7	653.5	1150.2
Variance:						
Constant	455.3***	121.5	449.1***	120.4	427.3***	133.8
Income	1.8***	0.7	1.8***	0.7	1.9**	0.8
Log pseudolikelihood	−6527.9		−6526.6		−6523.9	
*N*	1,080		1,080		1,080	

*Note*: Model [1]—no interaction terms; Model [2]—product and bidding round interaction; Model [3]—product and bidding round interaction, product and NERICA awareness interaction.

*p < 0.1; **p < 0.05; ***p < 0.0.

**Table 7.** Price premiums (discounts) for NERICA1 and NERICA4.

	**Price premium for *NERICA* relative to *Kaiso* (USh/kg)**		**Price premium/discount for *NERICA 1* relative to *Supa* (USh/kg)**	
	**NERICA 1**	**NERICA 4**	**Model [2]**	**Aware of NERICA - Model [3]**
Pooled	+522.2	+440.0		
Pre-tasting	+585.8	+497.5	−572 (1,163)	−591 (1,515)
Post-tasting	+481.7	+458.3	−694(1,228)	−716 (1,583)
Post-CIT	+499.2	+364.2	−528 (1,341)	−553 (1,695)

*Note*: Numbers in parentheses are (extrapolated) WTP values for *Supa* from Table 6. *Kaiso* is the benchmark variety; WTP values represents amounts to upgrade from *Kaiso.*

Beginning with the rice varieties, results show that relative to *Supa* (the superior quality reference category), participants were less likely to upgrade to NERICA4, an outcome which was statistically significant at the 1% level. Specifically, subjects were approximately 17 percentage points less likely to exchange *Kaiso* for NERICA4, compared with *Supa* (see Table 5). This lower level of preference for NERICA4 also translated into subjects’ WTP bids. Subjects, regardless of the bidding round, offered a premium of 519 USh/kg [= 1258 − 739] or US$ 0.20 for the variety (see Table 6 Model [1]). Preferences for NERICA1 were similar relative to *Supa*: Subjects were 14 percentage points less likely to upgrade the benchmark *Kaiso* to NERICA1 (Table 5), but paid a slightly higher premium of 661 USh/kg [= 1258 − 597], equivalent to US$ 0.26 (Table 6 Model [1]). It is apparent from these outcomes that, although the NERICAs were able to replace the inferior quality benchmark *Kaiso* in the non-fragrant rice category to some degree, they were far from being able to compete with the premium *Supa* variety in the fragrant category. The reasons for these variations in preferences, beyond the fragrance of *Supa*, could be further explored in future studies.

With respect to the experimental rounds, participants were more likely to upgrade to an alternative rice variety pre-tasting relative to post-tasting, by 16 percentage points. In contrast, subjects were only minimally disposed to upgrading post-CIT (Table 5). This outcome is consistent with Demont et al. (2013b) who observed a lower willingness to upgrade from a benchmark rice to an alternative after tasting. Xue et al. (2010) also reported decreased consumer preference for grass-fed beef after tasting.

Examination of the effect of bidding round on product specific WTP bids shows that NERICA1 was preferred over NERICA4 before tasting with average valuations of 591 USh/kg (US$ 0.23) and 372 USh/kg, (US$ 0.15) respectively (from Model 2).

The results also showed that having prior awareness of NERICA tended to influence WTP bids. From Table 6 Model [1], awareness of NERICA, in general, resulted in 306 USh/kg (US$ 0.12) increase in average WTP. Results extrapolated from Table 6 show that, awareness led to higher average valuation of both varieties across all bidding rounds, with average premiums ranging from 419 to 678 USh/kg (US$ 0.16 to US$ 0.27) for NERICA4, and from 867 to 1,142 USh/kg (US$ 0.34 to US$ 0.45) for NERICA1.

Other aspects of the experimental characteristics that influenced WTP bids included the time of the auction experiments. Results show that subjects who participated in the morning sessions were 8 percentage points more likely to switch from the benchmark *Kaiso* rice to an alternative rice. Such consumers were also willing to pay about 587 USh/kg (US$ 0.23) higher for the alternative rice than those who participated in the afternoon sessions. Similar findings were reported in Demont et al. (2012, 2013a) who argued that subjects who participated in the mornings likely had plans to purchase rice later in the day, and consequently valued the products higher than those who may have purchased rice before participating in afternoon experiments. Participants who reported being hungry during the experimental sessions were 7 percentage points less likely to exchange the benchmark rice for an alternative, although this did not have a significant impact on WTP bids. While the reason behind this is uncertain, participants in such physiological states may have been more likely than others to want to retain the cheaper and known *Kaiso* variety.

Demographic characteristics had a moderate impact on the probability of upgrading, and similarly, on WTP bids. Participants who engaged the services of cooking housemaids at home were less likely to upgrade to an alternative rice variety, by 14 percentage points. Very likely, such participants were not the most familiar with the different rice types, and perhaps preferred to stick with the benchmark variety. Participants with larger household sizes significantly discounted the alternative upgrades by about 123 USh/kg (US$ 0.05), consistent with our hypothesis, with this outcome likely due to economic concerns. Regarding ethnicity, participants from the dominant ethnic group, *Baganda*, were willing to pay between 936 to 941 USh/kg (US$ 0.37) more for an alternative rice type, depending on the model. However, this was surpassed by subjects who identified as *Acholi*; they were not only more likely to upgrade to an alternative rice, but they were willing to bid amounts between 1,580 USh to 1,610 USh/kg (US$ 0.62 to US$ 0.63) for an alternative rice. Participants from other ethnic groups such as *Banyagkole* and *Samia* were also willing to pay more for the alternative rice versions. These four ethnic groups all originate from an area that recently started growing rice and, as a result, are more exposed to the introduction of NERICA as a cash crop. Moreover, *Baganda* is concentrated around the city where *Supa* is the main traded local variety.

In modeling the variance portion (bottom of Table 6), only the income variable was found to be statistically significant and positive. This indicated high income variability with respect to WTP bids submitted for the alternative rice versions.

Beyond these, it was considered insightful to draw comparisons between both NERICAs based on their unique attributes. Specifically, given that the distinguishing attributes are fragrance versus non-fragrance, we compare NERICA1, the more fragrant of the two, to *Supa* in the fragrant rice category. Similarly, the non-fragrant NERICA4 is compared with *Kaiso* in the non-fragrant rice category. Table 7 displays price premiums for NERICA4 relative to *Kaiso*. Also shown are amounts indicating price discounts for NERICA1 with respect to *Supa*. Recall the experimental set-up requested bids to upgrade from *Kaiso* to the alternative varieties, suggesting that posted WTP values were price premiums over the benchmark *Kaiso*. For non-fragrant NERICA4, average price premiums ranged between 364 USh/kg and 497.5 USh/kg (i.e. US$ 0.14 and US$ 0.20). Examining this within the context of market prices provide a deeper perspective: 1 kg of *Kaiso* was priced anywhere between 2,800 USh and 3,000 USh (US$ 1.10 and US$ 1.12) in the Nakawa market at the time of the experiments, indicating that the price premiums for NERICA4 over *Kaiso* were between 12% to 17%.

Unlike the price premiums recorded for NERICA4 in the non-fragrant rice categories, the fragrant NERICA1 was discounted relative to the popular fragrant variety, *Supa*. Regardless of the rounds, NERICA1 was discounted between 39% to 57% relative to the fragrant *Supa*. These discounts persisted among participants who were familiar with NERICA, even if slightly lower (between 33% to 45%). Despite the agronomic novelty of NERICA, these results suggest they would under-compete with *Supa* among consumers more disposed to fragrant rice.

Even though both NERICAs were premium-priced over *Kaiso*, a comparison reveals that fragrance was the generally preferred attribute. In general, the price premium for the more fragrant NERICA1 was approximately 19% higher than the non-aromatic NERICA4. This would suggest a preference for NERICA1 between the two NERICA varieties. In fact, the discounts observed between NERICA1 and *Supa* reinforces participants predisposition for fragrant rice, with both NERICAs seemingly falling short of this expectation.

## Discussion and conclusion

To deliver genetic gains to rice farmers, scientists and investors are usually confronted with a dilemma of two seemingly aligned options. On the one hand, they can invest in breeding programs focused on delivering high-yielding varieties that increase farm productivity, enhance smallholders’ livelihoods, reduce food prices on the market, potentially diversify rice types, and hence foster consumer affordability and consumer choice. On the other hand, they can help smallholders gain access to pre-existing or new high-value markets by delivering varieties with premium traits that are highly demanded in the market and that fetch price premiums, and by doing so help them to increase incomes and their livelihoods.

We use the case of New Rice for Africa (NERICA) varieties to retrospectively assess the priorities breeders have adopted a decade and a half after these varieties were introduced. Market evidence from Uganda collected a decade after the release of NERICAs and before their popularity in urban markets suggests that consumers positioned the NERICAs between the standard non-fragrant, and premium, fragrant market standards: *Kaiso* and *Supa*, respectively. Consumers generally valued NERICA1 more than NERICA4, very likely, the result of its fragrance. What these outcomes reveal is that changing preferences about food is, perhaps, more nuanced when new products are introduced to compete with familiar ones. Study participants were more likely to exchange the inferior rice, *Kaiso*, for *Supa*, the known fragrant rice, than either NERICA4 or NERICA1. This was also consistent with the amounts they were willing to pay, which was higher for *Supa* than the NERICA varieties.

Although the 2011 market evidence would have suggested breeders prioritize investment in breeding programs for fragrant NERICAs to help smallholders gain access to high-value markets (see Table 7), the popularity of NERICA4 relative to NERICA1 in farmers’ fields a decade later appear to suggest that agronomic genetic gains may have outweighed market traits such as fragrance. This outcome shows that farmers and consumers alike viewed rice in terms of its varietal and attribute uniqueness. As results show, experimental participants were willing to pay price premiums for the non-fragrant NERICA4 over *Kaiso*, the inferior benchmark, an indication that among non-fragrant varieties, NERICA4 was valued higher. Given that NERICA4 is higher yielding and significantly reduces production costs, it can succeed as a cash crop and successfully increase household food security and health of farmers if the increased efficiency gains outweigh the loss in revenue due to price discounts on the market, relative to the most popular fragrant rice variety *Supa*. NERICA4 would then increase consumer choice and affordability by providing a cheaper, non-aromatic rice variety on the market for the urban poor that has superior characteristics than the prevailing market standard *Kaiso*. The present market reality of NERICA4 being blended and sold together with *Kaiso*, unfortunately suggests that farmers are not being rewarded for the superior quality characteristics of NERICA4, probably due to lack of branding, vertical coordination, and economies of scale. This means that there must be benefits for rice farmers in some other form, probably from the agronomic gains of NERICA4. While consumer choice may not necessarily have expanded for non-aromatic rice (given the two are comingled), the agronomic gains from NERICA4 would ultimately transmit to consumers through enhanced food security.

With respect to fragrance, the price discount observed for NERICA1 relative to *Supa* illustrates the challenge new varieties face as they compete with better known types within a premium quality class. This clearly reveals the disdain the former faces relative to the latter on the market. Thus, it may be argued that agronomic advantages may not be enough to turn the tide of consumer preferences if they are not at least as close in characteristics as other known/popular varieties. Consequently, the optimal strategy for breeders is to strike an optimal mix between both non-exclusive investment options, i.e. (i) high-yielding, non-aromatic NERICAs to increase farmer productivity and consumer affordability of rice; and (ii) fragrant NERICAs to increase farmers’ access to premium quality rice markets and enhance household food security.

Another outcome from study findings that should perhaps be of interest to policymakers is that participants who were aware of NERICA were willing to pay higher when they upgraded the benchmark rice to an alternative. Knowledge about the existence of NERICA supported results about the weak preference for NERICA4 among consumers sampled for the experiments; those who indicated some knowledge about the varieties valued NERICA4 less and instead, preferred NERICA1. While this suggests increasing consumer awareness about improved rice varieties could enhance preferences for them, such awareness would be expected to translate into weak consumer choice for less preferred varieties such as NERICA4, as the experimental evidence from 2011 showed. However, despite the lower initial demand signals from consumers, NERICA4 has become the most popular upland rice variety in Uganda, indicating that demand may not have been the overarching factor in smallholder farmers’ adoption decisions. It also shows that agronomic genetic gains may have outweighed genetic gains in market traits such as fragrance in the case of the Ugandan rice sector.

Important lessons can be gleaned from both experimental evidence in 2011 and the present market reality for NERICA in Uganda. First, while the goal of many genetically improved rice varieties is to invariably deliver productivity gains, breeders should also consider characteristics that consumers prefer. Similarly, genetically improved varieties may not necessarily supplant existing ones. For newly bred varieties to coexist with older ones, it is important that the improved variety, in addition to its agronomic advantages, mimics key attributes in existing varieties; fragrance, or non-aromatic attributes in this instance. In addition, where multiple varieties are concerned, it is crucial they are assessed against closely related existing varieties within their respective quality classes. Our findings would have suggested farmers prioritize fragrant NERICA1, given it fetched higher premiums over *Kaiso* than NERICA4, if the NERICAs were only evaluated against each other. However, evaluation within their respective fragrance categories would make a stronger case for NERICA4, which has been affirmed by the present market evidence.

Similar retrospective studies need to be conducted in other countries and for other crops. Breeders need to set agronomic priorities, based on imperfect information. Whenever they use market information, that information is in fact immediately outdated because breeding takes time. As a result, breeders risk consistently missing their targets given the challenge in predicting market trends a decade ahead. Therefore, we hope that retrospective studies like these can contribute to improved reflection and decision-making among breeders and breeding institutes around the world.

## Appendix

### Marginal WTP functions

#### Model [2]

Marginal WTP for NERICA4:

$$\frac{\partial WTP}{\partial NERICA4}=-590.5-200.6\text{Pre}\_\text{tasting}-256.1\text{Post}\_\text{CIT}$$

E.g. for post-tasting, the marginal WTP is computed as,

$${\frac{\partial WTP}{\partial NERICA4}|}_{Post\_taste=1}=-590.5$$

For pre-tasting,

$${\frac{\partial WTP}{\partial NERICA4}|}_{Pre\_taste=1}=-590.5-200.6=-791.1$$

etc.

Marginal WTP for NERICA1:

$$\frac{\partial WTP}{\partial NERICA1}=-694.1+121.9\text{Pre}\_\text{tasting}+166.6\text{Post}\_\text{CIT}$$

#### Model [3]

Marginal WTP for NERICA4:

$$\frac{\partial WTP}{\partial NERICA4}=-420.1-191.3\text{Pre}\_\text{tasting}-239.6\text{Post}\_\text{CIT}-484.4\text{aware}$$

Marginal WTP for NERICA1:

$$\frac{\partial WTP}{\partial NERICA1}=-680.9+125.2\text{Pre}\_\text{tasting}+163.8\text{Post}\_\text{CIT}-35.5\text{aware}$$
